# Role of SIK1 in the transition of acute kidney injury into chronic kidney disease

**DOI:** 10.1186/s12967-021-02717-5

**Published:** 2021-02-15

**Authors:** Jinxiu Hu, Jiao Qiao, Qun Yu, Bing Liu, Junhui Zhen, Yue Liu, Qiqi Ma, Yanmei Li, Qianhui Wang, Cheng Wang, Zhimei Lv

**Affiliations:** 1 Department of Nephrology, Shandong Provincial Hospital, Cheeloo College of Medicine, Shandong University, Jinan, 250021 Shandong China; 2 Department of Nephrology, Shandong Provincial Hospital Affiliated to Shandong First Medical University, Jinan, 250021 Shandong China; 3 Department of Pathology, School of Medicine, Shandong University, Jinan, 250021 Shandong China

**Keywords:** AA, SIK1, AKI-CKD transition, Wnt/β-catenin, Twist1

## Abstract

**Background:**

Acute kidney injury (AKI), with a high morbidity and mortality, is recognized as a risk factor for chronic kidney disease (CKD). AKI-CKD transition has been regarded as one of the most pressing unmet needs in renal diseases. Recently, studies have showed that salt inducible kinase 1 (SIK1) plays a role in epithelial-mesenchymal transition (EMT) and inflammation, which are the hallmarks of AKI-CKD transition. However, whether SIK1 is involved in AKI-CKD transition and by what mechanism it regulates AKI-CKD transition remains unknown.

**Methods:**

We firstly detected the expression of SIK1 in kidney tissues of AKI patients and AKI mice by immunohistochemistry staining, and then we established Aristolochic acid (AA)-induced AKI-CKD transition model in C57BL/6 mice and HK2 cells. Subsequently, we performed immunohistochemistry staining, ELISA, real-time PCR, Western blot, immunofluorescence staining and Transwell assay to explore the role and underlying mechanism of SIK1 on AKI-CKD transition.

**Results:**

The expression of SIK1 was down-regulated in AKI patients, AKI mice, AA-induced AKI-CKD transition mice, and HK2 cells. Functional analysis revealed that overexpression of SIK1 alleviated AA-induced AKI-CKD transition and HK2 cells injury in vivo and in vitro. Mechanistically, we demonstrated that SIK1 mediated AA-induced AKI-CKD transition by regulating WNT/β-catenin signaling, the canonical pathway involved in EMT, inflammation and renal fibrosis. In addition, we discovered that inhibition of WNT/β-catenin pathway and its downstream transcription factor Twist1 ameliorated HK2 cells injury, delaying the progression of AKI-CKD transition.

**Conclusions:**

Our study demonstrated, for the first time, a protective role of SIK1 in AKI-CKD transition by regulating WNT/β-catenin signaling pathway and its downstream transcription factor Twist1, which will provide novel insights into the prevention and treatment AKI-CKD transition in the future.

## Background

Acute kidney injury (AKI), a common disease characterized by a decrease in glomerular filtration rate (GFR) and an increase in serum creatinine [1], is regarded as a risk factor for the development and progression of CKD [2, 3]. Recently, AKI-CKD transition has become one of the hotspots in the study of kidney diseases. Although it is reported that inflammation, EMT, and fibrosis play a vital role in the progress of AKI-CKD transition [4, 5], the exact molecular mechanism of AKI-CKD transition is still unclear.

Salt Inducible Kinase 1 (SIK1) is a member of the AMP-activated protein kinases (AMPKs) family, which play pivotal roles in regulating metabolism, cell survival, and growth [6, 7]. Studies have demonstrated that inhibition of SIK1 promotes EMT, leading to migration and metastasis of tumors [8–10]. Besides, it was reported that SIK1 negatively regulate the TLR4-induced activation of NF-κB and attenuated expressions of proinflammatory cytokines [11]. In addition, emerging evidences showed a link between SIK1 and kidney diseases [6, 12]. For instance, upregulation of SIK1 reversed the high glucose-induced mesangial cells proliferation, and extracellular matrix (ECM) accumulation by inhibiting the expression of FN and PAI-1, both of which are involved in fibrotic disorders, such as glomerulosclerosis [6, 13]. Thus, we speculate that SIK1 might be involved in the progression of AKI-CKD transition, which is characterized with EMT, inflammation and renal fibrosis.

WNT/β-catenin pathway is a complex, highly conserved signaling pathway that regulates various biologic processes, such as organ development, tissue homeostasis and carcinogenesiss [14, 15]. Although silent in the normal adult kidney, it is found to be reactivated in a variety of kidney diseases, including acute kidney injury, diabetic nephropathy, interstitial fibrosis and cystic kidney diseases [16]. Moreover, it is reported that silencing of WNT/β-catenin pathway ameliorates renal fibrosis, delaying the progression of AKI-CKD in I/R-induced injury [17]. But whether SIK1 could participate in the AKI-CKD transition by modulating WNT/β-catenin pathway remains to be further clarified.

Emerging evidences have shown that Snail and Twist1, the EMT transcription factors, play important roles in the pathogenesis of renal fibrosis [18–20]. It was reported that conditional deletion of Twist1 or Snail in proximal tubular epithelial cells inhibited EMT, attenuated interstitial fibrosis in experimentally induced renal fibrosis in mouse [21]. Considering Snail and Twist1 are significant molecules located in the downstream of WNT/β-catenin pathway, we speculated that they may also played a vital role in SIK1 mediated AKI-CKD transition.

Thus, in this study, we aimed to elucidate the role and mechanism of SIK1 in AKI-CKD transition, which will provide a new therapeutic target for clinical prevention and treatment of renal fibrosis and will provide a new way to delay the progress of AKI-CKD transition.

## Materials and methods

### Patients and tissue samples

The study was approved by the Clinical Research Ethics Committee of Shandong Provincial Hospital Affiliated to Shandong University. Human kidney samples were collected from patients with AKI diagnosed based on renal biopsy, and the kidney samples in the control groups were obtained from paracancerous tissue of patients with kidney tumors who underwent surgical resection. The tissue samples were preserved in liquid nitrogen and the informed consent was signed by all patients in accordance with the Declaration of Helsinki.

### Construction of AAV9-*Sik1*

Recombinant adeno-associated virus (AAV9-*Sik1*) and adeno-associated virus negative control (AAV9-*NC*) were constructed by GENECHEM (Shanghai, China). Recombinant AAV9-*Sik1* adeno-associated virus vector GV467 includes promoter, antibody coding region, EGFP green fluorescent protein coding region, and 3FLAG tag protein coding region. The ability of AAV9 vector to transduce kidney has been verified (Additional file 1).

### Animals and modeling methods

Modeling and follow-up experimental programs have been approved by the Animal Care and Use Committee of Shandong University. C57BL/6 mice aged 4–6 weeks and weighing 15–20 g were supplied by the Experimental Animal Center of Shandong University and were randomly allocated into 4 groups: control group (Control), normal mice treated with AA group (AA), AA mice treated with AAV9-*Sik1* group (AAV9-*Sik1* + AA), AA mice treated with AAV9-NC group (AAV9-*NC* + AA). The mice in AA group were injected with AA (10 mg/kg) intraperitoneally, while the mice in control group were injected with PBS. On the 3rd, 7th, 14th, and 28th after AA injection, the 24 h urine of mice was collected in a metabolic cage and the supernatant was stored at − 20 °C after centrifugation. Blood was centrifuged at 3000r for 10 min, and the supernatant was stored at − 20 °C. 24 h urinary protein, serum creatinine (Scr), and blood urea nitrogen (BUN) were measured by automatic bio-chemical analyzer. Serum IL-1β and TNF-α were detected by ELISA. Then, the mice were sacrificed and weighed. After that kidneys were excised and weighed. All kidney samples were divided into two parts, one of which was fixed in 4% paraformaldehyde for histological staining, while the other was stored under − 80 °C for real-time PCR and Western blot detection.

### Histology and immunohistochemistry

After fixing in 4% paraformaldehyde, the kidney samples were dehydrated, transparent, embedded in paraffin and sectioned into 4 µm‑thick slices. Subsequently, the slices were dewaxed, hydrated and stained. For renal histological analysis, HE staining, PAS staining and Masson's trichrome staining were used according to the manufacturer’s instructions. For immunohistochemical analysis, sections were incubated with primary antibody against SIK1(51045-1-AP, Proteintech, USA), E-cadherin (20874-1-AP, Proteintech, USA), α-SMA (ab32575, Abcam, USA), COL1 (bs-10423R, Bioss, Beijing, China) at 4 ℃ overnight. After incubation with secondary antibody, the DAB was added, followed by nuclear counterstaining with hematoxylin. Finally, the images were observed under microscope (Leica, Germany). The percentage of positive staining for SIK1, E-cadherin, α-SMA, and COL1 was measured by using a quantitative Image-Pro plus 6.0 software (Media Cybernetics, Silver Spring, MD) [22].

### Cell culture and treatment

Human proximal tubular epithelial cells (HK2) were cultured in DMEM medium (Gibco, USA) supplemented with 10% fetal bovine serum (FBS) (Gibco, USA) and 1% penicillin–streptomycin (Sigma, USA) at 37 °C in a humidified atmosphere containing 5% CO_2_. Aristolochic acid (AA) (Sigma, USA) was dissolved in DMSO to 1 mg/mL and cells were treated with the final concentration at 10 µmol/L, 20 µmol/L, 40 µmol/L and 60 µmol/L, and the control group was treated with the same amount of DMSO.

### Lentiviral vector transduction and siRNA transfection

The lentiviral shRNA constructs targeting *SIK1*, β-catenin and a scrambled shRNA, as well as lentiviral overexpression vector for *SIK1* and empty control vector were constructed by Cyagen (Guangzhou, China). The target sequences are listed as follows: *SIK1* shRNA: 5′-GCGCGTGCATTGATTACTATC-3′; β-catenin shRNA: 5′-TTTGATCCCATCTTCCGCAGC-3′; Scramble shRNA: 5′-CCTAAGGTTAAGTCGCCCTCG-3′. The lentivirus infection was conducted following the standard protocol provided by the manufacturer. *Twist1* siRNA and a negative control sequence (NC siRNA) were designed by RiboBio (Guangzhou, China). The target sequences were listed as follows: *Twist1* siRNA: 5′-CCGGAGACCTAGATGTCAT-3′; NC siRNA: 5′-UUCUCCGAACGUGUCACGUTT-3′. Transfection of HK2 cells was performed in a 6-well plate using Lipofectamine^TM^2000 (Invitrogen, USA) according to the manufacturer’s instructions.

### Cell counting kit-8 (CCK-8)

Cells were seeded in a 96-well plate at a density of 2 × 10^4^/mL and then treated with different concentrations of AA (10, 20, 40, 60 µmol/L) in an incubator containing 5% CO_2_ at 37 ℃. After 72 h treatment, 10μL of CCK-8 solution (YESEN, Shanghai, China) was added into each well. After incubation for 1.5 h, the absorbance at 450 nm of each well was measured with a microplate reader (Thermo Fisher Scientific, USA). Five replicated wells were used for different groups and experiments were performed in triplicate.

### Enzyme-linked immunosorbent assay (ELISA)

The concentrations of IL-1β and TNF-α in the serum or in the supernatant of treated HK2 cells were collected and detected using ELISA Kit (ColorfulGene, Wuhan, China) according to the manufacturer’s instructions. The absorbance O.D. was read at 450 nm using a Microtiter Plate Reader.

### RNA extraction and quantitative real-time PCR analysis

Total RNA was extracted from HK2 cells or kidney tissues using TRIzol reagent (Takara, Japan). RNA purity was assessed using a NanoDrop-2000 spectrophotometer (Thermo Fisher Scientific, USA) and each RNA sample had an A260:A280 above 1.8 and A260:A230 above 2.0. Subsequently, 1.0 μg RNA from each sample were reverse transcribed using the PrimeScript™ RT Reagent Kit (Takara, Japan) according to the manufacturer’s instructions and PCR amplification was performed using SYBR^®^ Premix Ex Taq (Takara, Japan) in the LightCycler^®^ 480 Real-Time PCR system (Roche, USA). The sequences of primers used in qPCR were listed in Additional table. β-actin was used as the internal control and the relative expression levels were calculated using the 2^−∆∆CT^ method. All the reactions were repeated in triplicate.

### Western blot analysis

After cells or kidney tissues were fully lysed with RIPA lysate (Beyotime, China) containing protease inhibitor and phosphorylated protease inhibitor, the protein was denatured. Then about 20 μg of protein was separated using SDS-PAGE and transferred to PVDF membranes (Millipore, USA). Subsequently, the membranes were blocked in 5% non-fat milk powder or 5% BSA for 1 h at room temperature in order to break non-specific binding, followed by incubation with primary antibody against SIK1 (51045-1-AP, Proteintech, USA), Caspase1/p20/p10 (22915-1-AP, Proteintech, USA), E-cadherin (20874-1-AP, Proteintech, USA), Vimentin (60330-1-Ig, Proteintech, USA), p-SIK1(Thr182)(PA5-105918, Thermo Fisher Scientific, USA), WNT1(ab15251, Abcam, USA), β-catenin(ab32572, Abcam, USA), p-β-catenin(Y654) (ab59430, Abcam, USA), ZO-1(ab96587, Abcam, USA), α-SMA(ab32575, Abcam, USA), Fsp1(ab197896, Abcam, USA), FN(ab2413, Abcam, USA), β-actin(ab8227, Abcam, USA), Histone 3(ab176842, Abcam, USA), COL1 (bs-10423R, Bioss, Beijing, China), Snail(#3879, Cell Signaling Technology, USA), and Twist1(#46702, Cell Signaling Technology, USA) at 4 ℃ overnight. After incubation with horseradish peroxidase-conjugated goat anti-rabbit IgG (ab6721, Abcam, USA) or horseradish peroxidase-conjugated goat anti-mouse IgG (ab6728, Abcam, USA) for 1 h at room temperature, the results were analysis using ECL reagent (Millipore, USA) and Amersham Imager 600 (GE, USA). β-actin was used as an internal control of total proteins, and Histone 3 was used as an internal control of nuclear proteins.

### Immunofluorescence staining

Cells were plated in 24-well slides followed by different interventions and then fixed with 4% paraformaldehyde. After penetration with 0.3% Triton X-100, the cells were incubated with 5% BSA for 30 min at room temperature. Next, the cells were incubated with β-catenin, E-cadherin, and Vimentin antibody at 4 °C overnight. Subsequently, Alexa Fluor^®^ 594-conjugated donkey anti-rabbit IgG (ab150076, 1:200, Abcam, USA) and Alexa Fluor^®^ 594-conjugated goat anti-mouse IgG (ab150116, 1:200, Abcam, USA) antibodies were applied for 1 h at 37 ℃ protected from light. After incubation with DAPI for 10 min, the cells were observed with a fluorescence microscope (Leica, Germany).

### Transwell migration assay

For the Transwell assay, 1–2 × 10^4^/mL HK2 cells in 150 μL serum-free medium were added to the upper chamber of Transwell and 600 μL DMEM medium containing 10% FBS were added to the lower chamber. Next, the cells were incubated at 37 ℃ for 24 h, and then fixed with 4% paraformaldehyde, and penetrated with 0.3% Triton X-100. After that, the non-invading cells were removed, the invaded cells were dyed with hematoxylin and then counted under a light microscope (Olympus, Japan).

### Statistical analysis

GraphPad Prism 8 and Adobe Photoshop were used to analyze experimental data and draw statistical graphs. The results were shown as the mean ± s.d. Statistical significance between two groups was determined by Student t-test while significance among multiple groups was determined by one-way ANOVA. *P* < 0.05 indicated a statistically significant difference. All experiments were repeated at least three times unless otherwise stated.

## Results

### SIK1 was down-regulated in AKI

**Fig.1:**
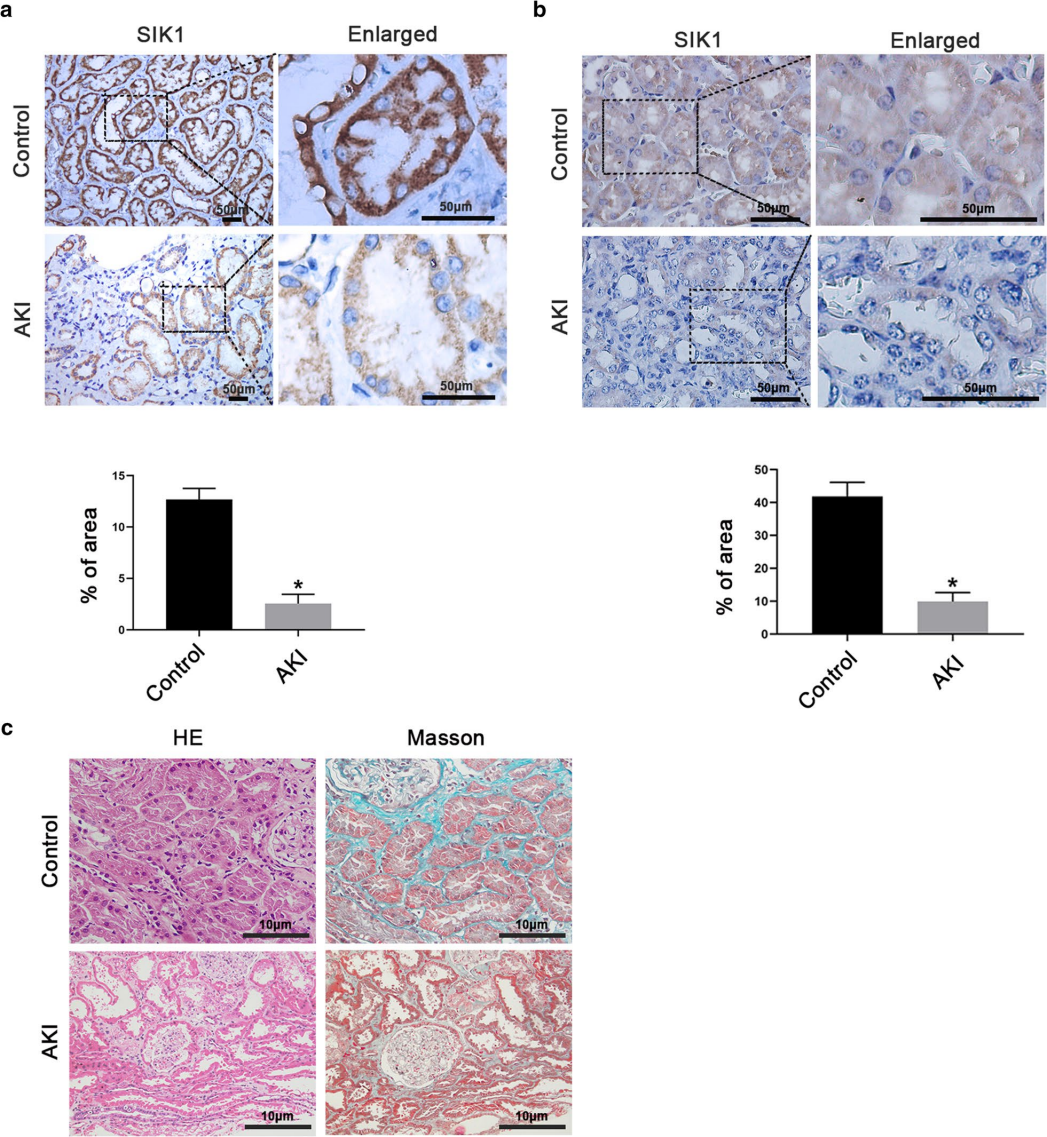
SIK1 was down-regulated in AKI. **a** Representative immunohistochemical staining images of SIK1 in Control and AKI patients. Scale bar = 50 μm. The graph showed the quantitative analysis of SIK1 in immunohistochemically stained sections. **b** Representative immunohistochemical staining images of SIK1 in Control and AKI mice. Scale bar = 50 μm. The graph showed the quantitative analysis of SIK1 in immunohistochemically stained sections. **c** Representative images of HE and Masson's trichrome staining in kidney tissues of AKI patients. Scale bar = 10 μm. Data are shown as mean ± s.d. *P < 0.05 vs Control. All experiments were performed in triplicate

Considering that SIK1 plays a significant role in kidney injury, we firstly detected the expression of SIK1 in kidney tissues of AKI patients and AKI mice. Immunohistochemistry staining revealed that, not only in patients but in mice, the SIK1 expression of renal tubules in control is higher than that in AKI group, which indicated that SIK1 was down-regulated during AKI (Fig. 1a, b). Besides, we conducted HE and Masson's trichrome staining to analyze the histopathological changes in kidney samples of AKI patients (Fig. 1c). Compared with control, renal tubular epithelial cells swelling, vacuolar degeneration, and interstitial edema were observed in the AKI group by HE staining. Moreover, compared with control, notably flattened tubular cells, detached brush border, and enlarged tubular limens were observed in AKI group by Masson's trichrome staining. Taken together, all these findings indicated that SIK1 might play a role in tubular injury.

### SIK1 was down-regulated in AA-induced AKI-CKD transition mice

**Fig.2:**
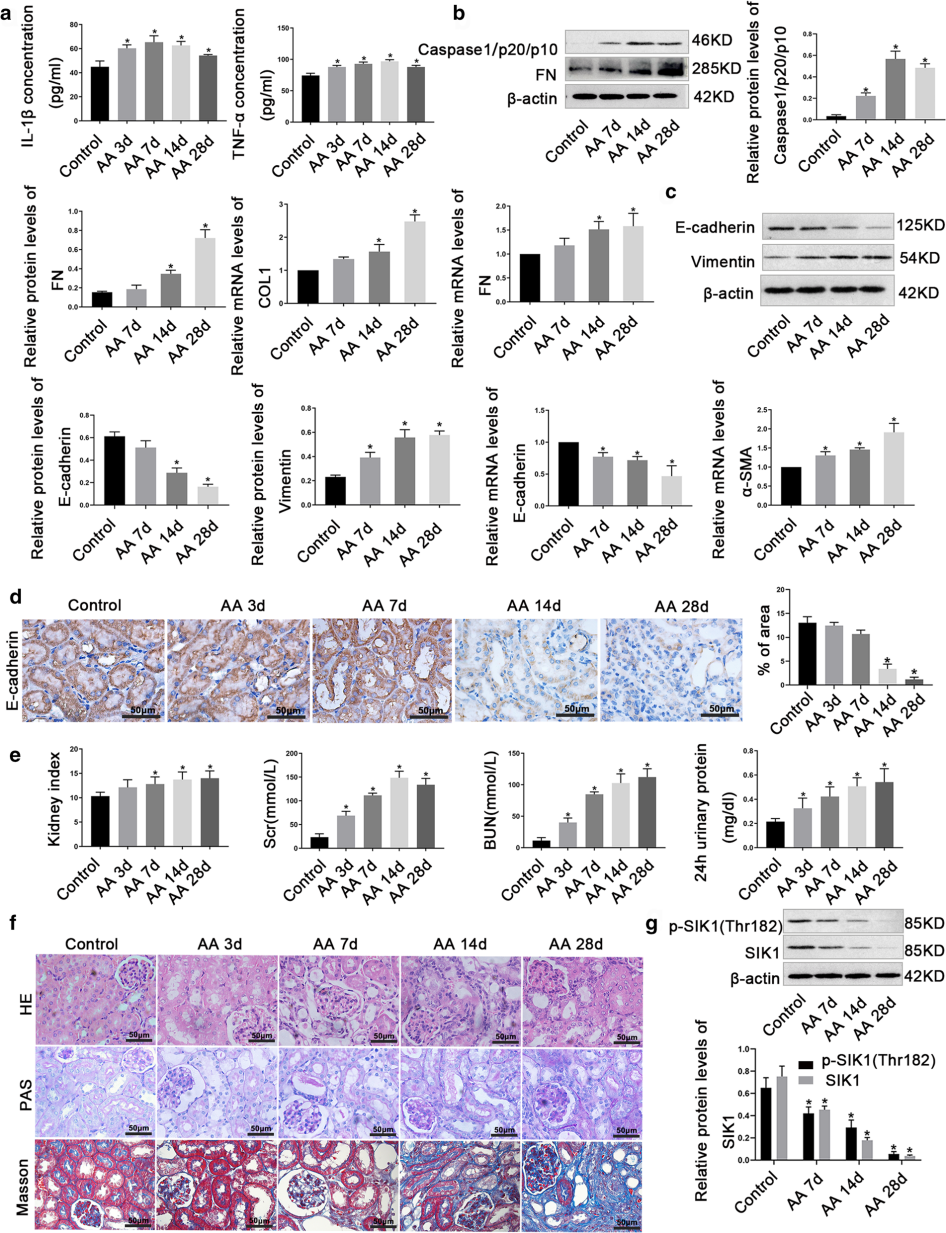
SIK1 was down-regulated in AA-induced AKI-CKD transition mice. **a** ELISA analysis of IL-1β, and TNF-α expression in different groups of mice. **b** Western blot and real-time PCR analysis of inflammation and fibrosis markers (Caspase1/p20/p10, FN and COL1) expression; **c** Western blot and real-time PCR analysis of EMT markers (E-cadherin, Vimentin, and α-SMA) expression. **d** Representative immunohistochemical staining images of E-cadherin in different groups of mice. Scale bar = 50 μm. The graph showed the quantitative analysis of E-cadherin in immunohistochemically stained sections. **e** The kidney index, Scr, BUN and 24 h urinary protein levels in different groups of mice. **f** Representative histological staining (HE, PAS, and Masson's trichrome staining) images of kidney tissues in different groups of mice. Scale bar = 50 μm. **g** Western blot analysis of SIK1 and p-SIK (Thr182) protein levels in C57BL/6 mice. Data are shown as mean ± s.d. *P < 0.05 vs Control. All experiments were performed in triplicate

To obtain a mice model of AKI-CKD transition, we injected AA intraperitoneally into mice. Compared with control, the mice treated with AA exhibited increased production of inflammatory factors, EMT and renal fibrosis (Fig. 2a–d). In addition, the kidney index, serum Scr, BUN and 24 h urinary protein enhanced upon AA treatment (Fig. 2e). Furthermore, AA impaired renal structure, leading to renal tubular epithelial cells atrophy, limen enlargement, and different extent of collagen fiber deposition in tubulointerstitium (Fig. 2f). All above indicated that the AA-induced AKI-CKD transition model was successfully established. To explore the role of SIK1 in AA-induced AKI-CKD transition, we assessed its expression in the samples of mice kidney injected with AA. We found SIK1 and p-SIK1 (Thr182) were decreased in a time-dependent manner after AA injection (Fig. 2g), which indicating a potential role for SIK1 in regulating AA-induced AKI-CKD transition.

### Overexpression of SIK1 alleviated AA-induced AKI-CKD transition

**Fig. 3:**
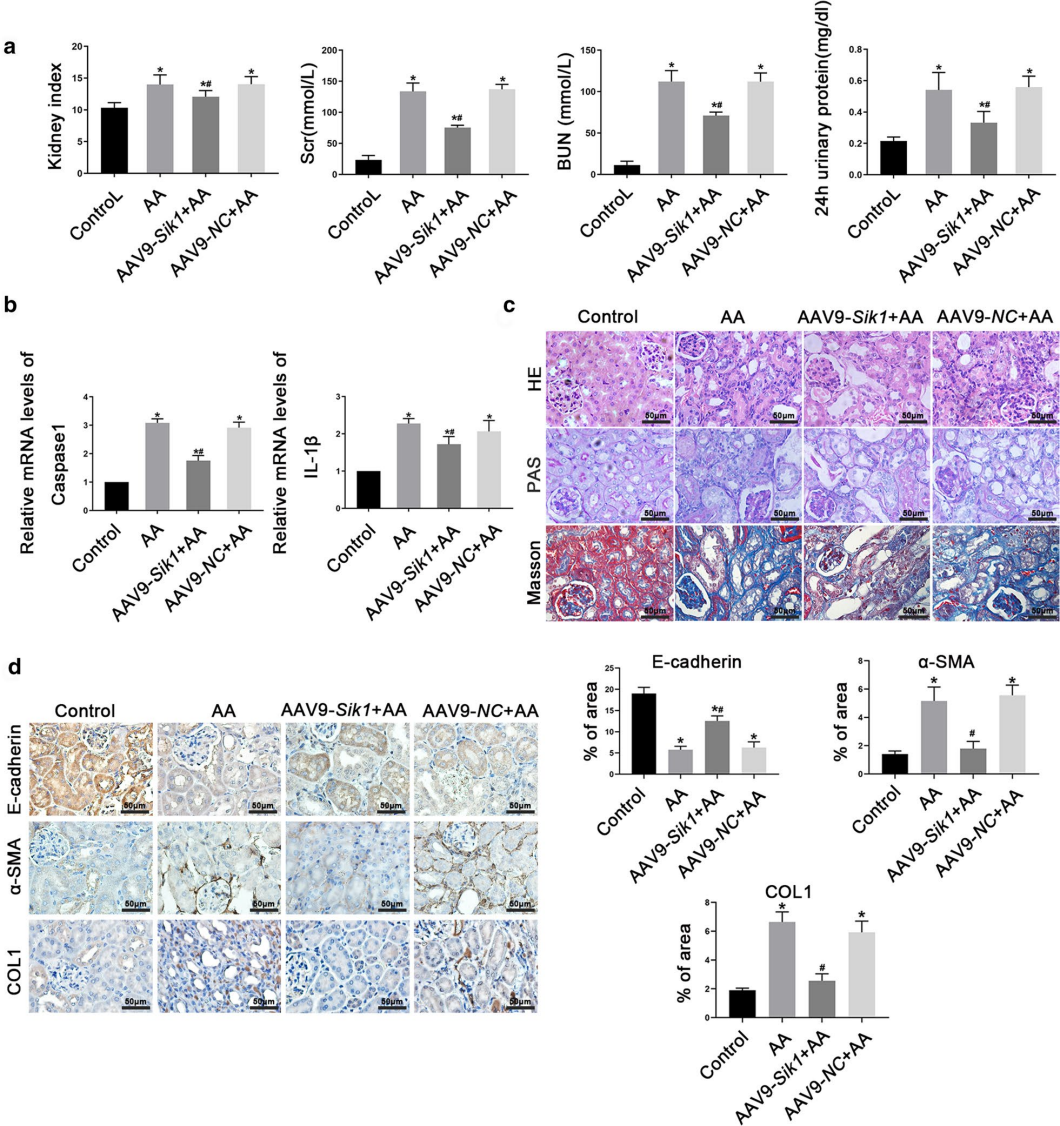
Overexpression of SIK1 alleviated AA-induced AKI-CKD transition. **a **The kidney index, Scr, BUN and 24 h urinary protein levels in different groups of mice. **b** real-time PCR analysis of Caspase 1, and *IL-1β* mRNA levels in different groups of mice. **c** HE, PAS and Masson's trichrome staining were used to assess the histological changes and the extent of tubulointerstitial fibrosis. Scale bar = 50 µm. **d** Representative immunohistochemical staining images of E-cadherin, α-SMA, and COLI protein in different groups of mice. Scale bar = 50 µm. The graphs showed the quantitative analysis of E-cadherin, α-SMA, and COL1 in immunohistochemically stained sections. Data are shown as mean ± s.d. *P< 0.05 vs Control, ^#^*P*< 0.05 vs AA. All experiments were performed in triplicate

To specify the function of SIK1 in AA-induced AKI-CKD transition, we injected AAV9-*Sik1* into tail vein of mice to overexpress SIK1. We found after injected with AAV9-*Sik1*, AA-induced renal dysfunction was significantly ameliorated, as evidenced by reduced levels of kidney index, Scr, BUN and 24 h urinary protein (Fig. 3a). Real-time PCR revealed that AAV9-*Sik1* relieved AA-induced inflammatory response (Fig. 3b). In addition, AAV9-*Sik1* significantly improved the histopathological damage induced by AA (Fig. 3c). Furthermore, the results of immunohistochemical staining suggested that AAV9-*Sik1* alleviated interstitial fibrosis and EMT in the process of AA-induced AKI-CKD transition (Fig. 3d). Overall, these results indicated that SIK1 played a protective role in AA-induced AKI-CKD transition.

### SIK1 was down-regulated in AA-treated HK2 cells

**Fig.4:**
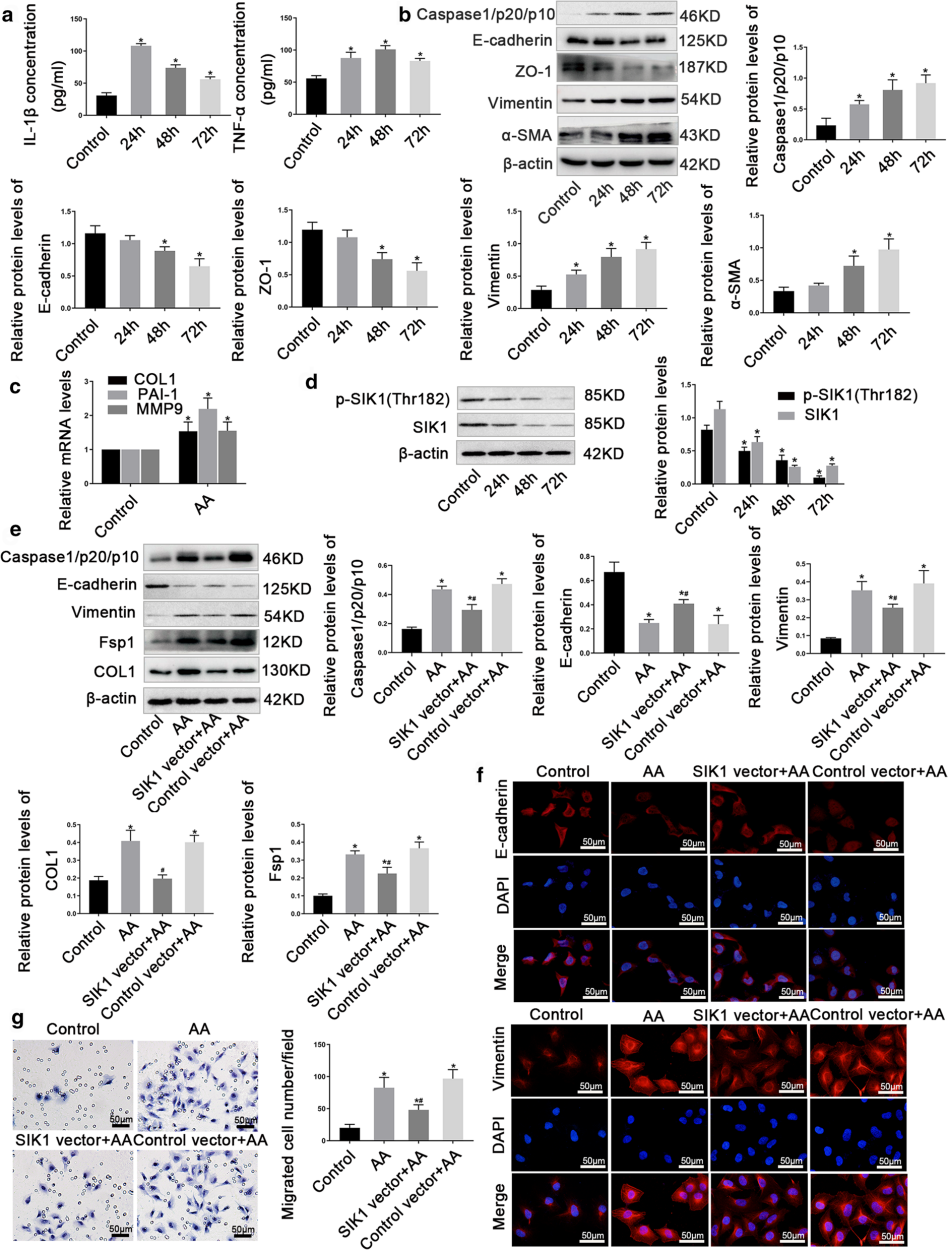
SIK1 was down-regulated in AA-treated HK2 cells and overexpression of SIK1 improved AA-induced HK2 cells injury. **a** ELISA detection of IL-1β and TNF-α levels in the supernatant of HK2 cells treated with 10 μmol/L AA for 0 h, 24 h, 48 h and 72 h. **b** Western blot analysis of Caspase 1/p20/p10, E-cadherin, ZO-1, Vimentin, and α-SMA protein levels in HK2 cells treated with 10 μmol/L AA for 0 h, 24 h, 48 h and 72 h, with the greatest effect after 72 h of treatment. β-actin was used as a control. **c** real-time PCR analysis of early fibrosis indicators (*COLI, PAI-1*, and *MMP9*) mRNA levels in HK2 cells treated with 10 μmol/L AA for 72 h. **d** Western blot analysis of p-SIK1(Thr182) and SIK1 levels in HK2 cells treated with 10 μmol/L AA for 0 h, 24 h, 48 h and 72 h. **e** Western blot analysis of Caspase1/p20/p10, E-cadherin, Vimentin, Fsp1, and COL1 in HK2 cells that are treated with SIK1 vector (SIK1 lentiviral overexpression vector) in the presence of 10 µmol/L AA or treated with 10 µmol/L AA alone for 72 h. **f** Representative immunofluorescence images of E-cadherin and Vimentin in HK2 cells. Scale bar = 50 μm. **g** Representative migration results of HK2 cells. Scale bar = 50 μm. Data are shown as mean ± s.d. *P < 0.05 vs Control, ^#^*P* < 0.05 vs AA. All experiments were performed in triplicate

A large number of studies have shown that the proximal tubule of the kidney is one of the main targets of injury in AKI, and the injury of proximal tubule may play an important pathophysiologic role in the development of AKI-CKD transition. To further assess whether SIK1 was down-regulated in vitro, we focused on HK2 cells in this study. By performing CCK8 assays, we choose 10 µmol/L AA as the optimal concentration for subsequent experiments (Additional file 2). Consistently, AA treatment exhibited increased inflammation, EMT and fibrosis in HK2 cells (Fig. 4a–c), suggesting AA induced HK2 cells injury in vitro. Subsequently, we examined the protein levels of SIK1 and observed a notably decreased SIK1 and p-SIK1 (Thr182) in HK2 cells in the presence of AA (Fig. 4d). Furthermore, we detected the location of SIK1 protein in HK2 cells before and after exposure to AA by Immunofluorescence staining. In the unstimulated cells, SIK1 was observed in both the nucleus and cytoplasm. When treated with AA, the expression of SIK1 was reduced and the SIK1 was gradually detected in the nucleus (Additional file 3). Collectively, these results elucidated that SIK1 was involved in AA-induced HK2 cells injury.

### Overexpression of SIK1 improved AA-induced HK2 cells injury

To further elucidate the role of SIK1 in AA-induced HK2 cells injury, we constructed cell lines that stably up-regulated SIK1 by lentivirus infection of HK2 cells (Additional file 4). Overexpression of SIK1 led to a significantly increased E-cadherin and repressed Caspase1/p20/p10, Vimentin, COLI, and Fsp1, when compared with the control cells stimulated with AA alone (Fig. 4e, f). Besides, overexpression of SIK1 inhibited the migration ability of HK2 cells induced by AA (Fig. 4g). Together, these findings demonstrated that SIK1 was protective against AA-induced injury in HK2 cells.

### SIK1 regulated WNT/β-catenin signaling pathway in vivo and in vitro

**Fig.5:**
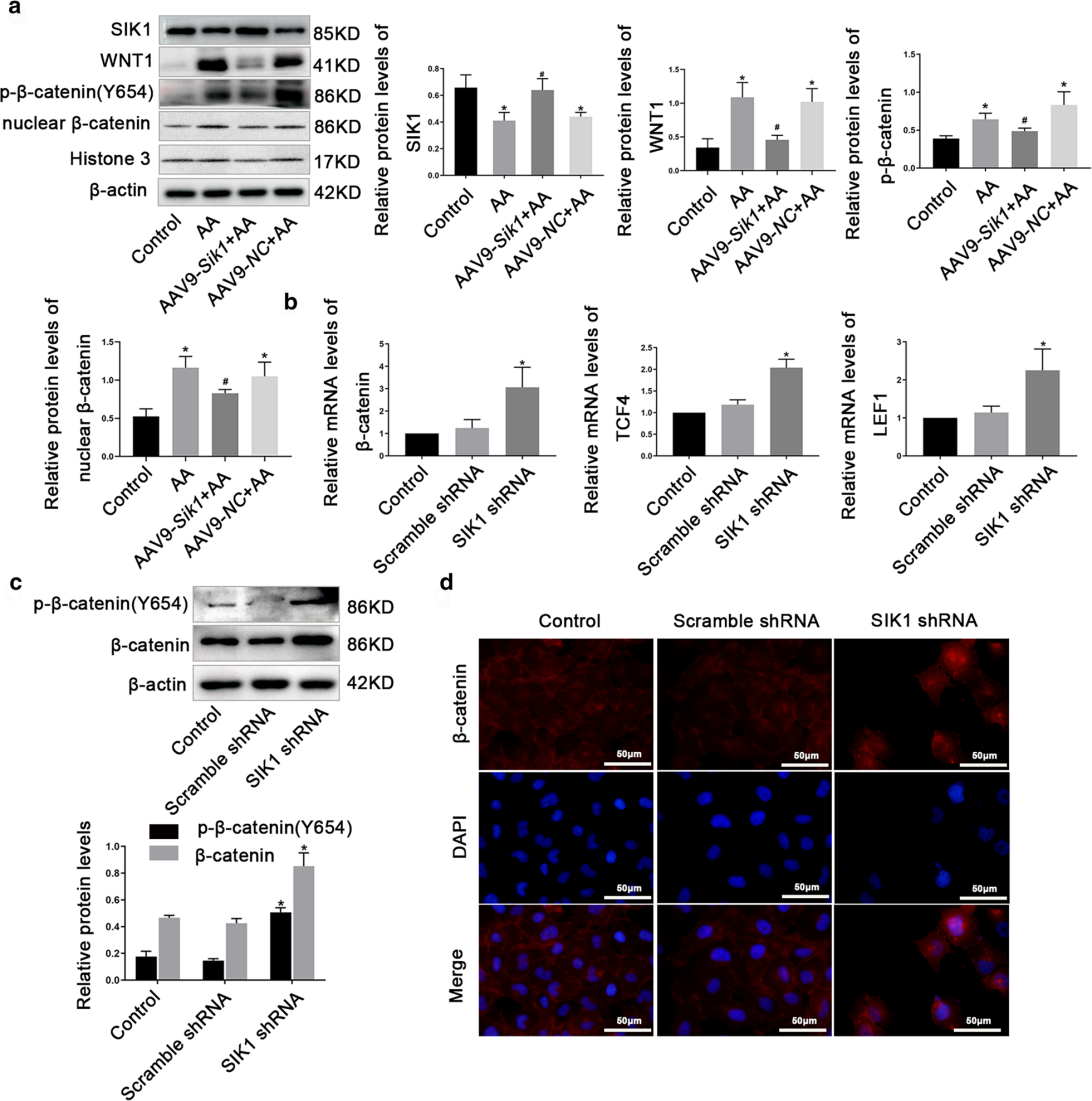
SIK1 regulates WNT/β-catenin pathway in vivo and in vitro. **a** Western blot analysis of SIK1, WNT1, p-β-catenin (Y654) and nuclear β-catenin protein levels in different groups of mice. **b** real-time PCR analysis of β-catenin, TCF4 and LEF1 in HK2 cells treated with *SIK1* shRNA or Scramble shRNA. **c** Western blot analysis β-catenin, and p-β-catenin(Y654) in HK2 cells treated with *SIK1* shRNA or Scramble shRNA. **d** Representative immunofluorescence images of β-catenin in HK2 cells. Scale bar = 50 μm. Data are shown as mean ± s.d. *P < 0.05 vs Control, ^#^*P* < 0.05 vs AA. All experiments were performed in triplicate

Considering the critical role of the WNT/β-catenin pathway in AKI-CKD transition, we explored whether SIK1 regulated WNT/β-catenin pathway. In vivo experiments, we observed that overexpression of SIK1 inhibited the protein levels of WNT1, p-β-catenin (Y654), and nuclear β-catenin in AA-induced AKI-CKD mice (Fig. 5a). To further explore whether SIK1 regulated WNT/β-catenin pathway in vitro, we silenced the expression of SIK1 by shRNA in HK2 cells (Additional file 5). Consistent with the in vivo results, silencing of SIK1 resulted in a notably increased β-catenin, *TCF4* and *LEF1* mRNA levels (Fig. 5b). Besides, knockdown of SIK1 increased the protein levels of β-catenin and p-β-catenin (Y654) (Fig. 5c). In addition, knockdown of SIK1 promoted the nuclear translocation of β-catenin (Fig. 5d). Collectively, these data suggested that SIK1 regulated WNT/β-catenin pathway in vivo and in vitro.

### WNT/β-catenin signaling pathway is involved in AA-induced HK2 cells injury

**Fig.6:**
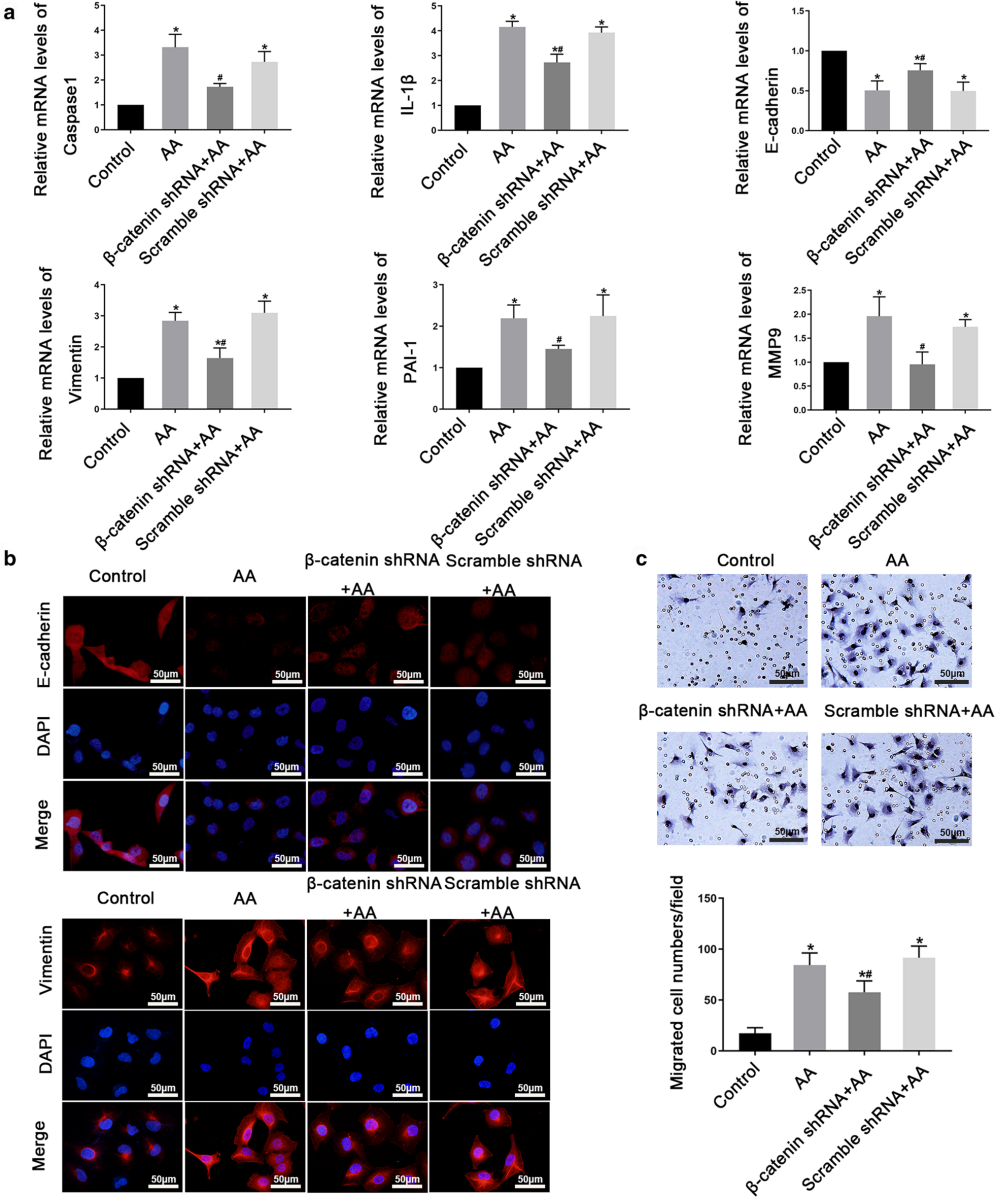
WNT/β-catenin signaling pathway is involved in AA-induced HK2 cells injury. HK2 cells were treated with β-catenin shRNA or Scramble shRNA in the presence with 10 µmol/L AA. **a** real-time PCR analysis of Caspase1, *IL-1β*, E-cadherin, Vimentin, *PAI-1*, and *MMP9* mRNA levels in HK2 cells. **b** Representative immunofluorescence images of E-cadherin and Vimentin in HK2 cells. Scale bar = 50 μm. **c** Representative migration results of HK2 cells. Scale bar = 50 μm. Data are shown as mean ± s.d.*P < 0.05 vs Control, ^#^ *P* < 0.05 vs AA. All experiments were performed in triplicate

To explore whether WNT/β-catenin pathway played a role in AA-induced HK2 cells injury, we tested the expression levels of WNT1, nuclear β-catenin, and p-β-catenin (Y654) after AA treatment. Results of Western blot showed that WNT1, nuclear β-catenin and p-β-catenin (Y654) increased significantly after AA stimulation (Additional file 6a). Besides, immunofluorescence staining revealed the nuclear translocation of β-catenin (Additional file 6b), which suggesting that AA stimulation can activate WNT/β-catenin signaling pathway. Considering β-catenin is the central component of WNT/β-catenin pathway, we wonder whether regulation of β-catenin regulates AA-induced HK2 cells injury. Thus, we stably knocked down β-catenin by shRNA lentivirus in HK2 cells (Additional file 6c). And we observed that β-catenin knockdown impaired the AA-induced Caspase1, IL-1β, Vimentin, PAI-1, and MMP9 expression and promoted E-cadherin expression (Fig. 6a, b). Moreover, β-catenin shRNA cells stimulated with AA exhibited significantly decreased migration compared with the β-catenin control cells stimulated with AA alone (Fig. 6c). Taken together, these findings suggested that WNT/β-catenin pathway was involved in AA-induced HK2 cells injury.

### WNT/β-catenin signaling pathway is required for SIK1 mediated HK2 cells injury induced by AA

**Fig. 7:**
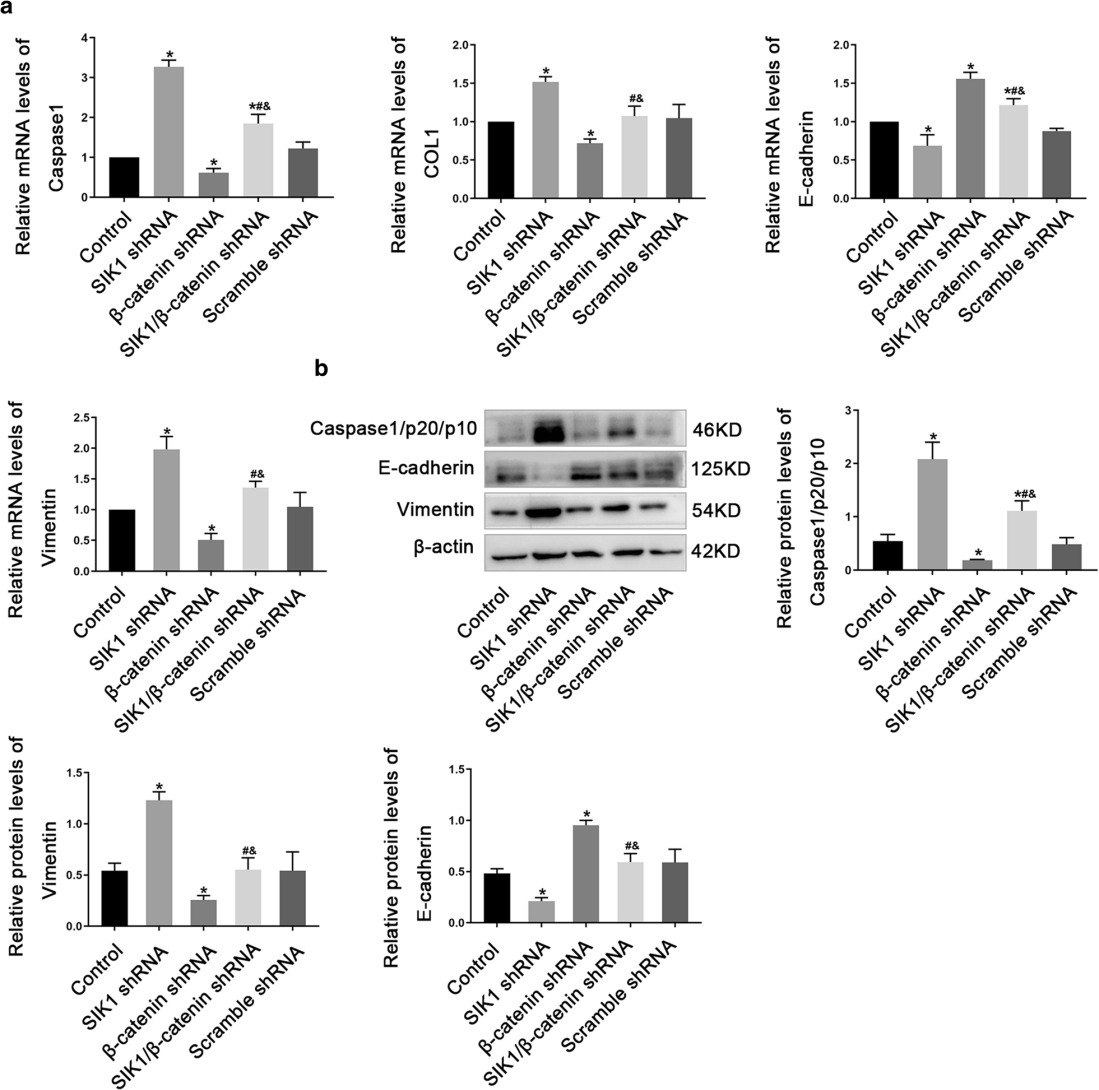
WNT/β-catenin pathway is required for SIK1 mediated HK2 cells injury induced by AA. HK-2 cells were co-transfected with *SIK1* and β-catenin shRNA or transfected with *SIK1* or β-catenin shRNA alone, and then treated with 10 µmol/L AA for 72 h. **a** real-time PCR analysis of Caspase1, COL1, E-cadherin, and Vimentin expression. **b** Western blot analysis of Caspase1/p20/p10, E-cadherin, and Vimentin expression. Data are shown as mean ± s.d.*P < 0.05 vs Control, ^**#**^*P* < 0.05 vs SIK1 shRNA, ^&^*P* < 0.05 vs β-catenin shRNA. All experiments were performed in triplicate

To study whether SIK1 regulated AA-induced HK2 cells injury through WNT/β-catenin signaling pathway, β-catenin was further decreased in SIK1 knockdown HK2 cells. Compared with SIK1 knockdown cells, further knockdown of β-catenin decreased the mRNA levels of Caspase1, COL1 and Vimentin, while increased the mRNA levels of E-cadherin (Fig. 7a). The results of Western blot were consistent with real-time PCR, showing silencing of β-catenin and SIK1 reversed the downregulation of E-cadherin, upregulation of Caspase1/p20/p10 and Vimentin induced by SIK1 knockdown (Fig. 7b). Collectively, these results indicated that inhibition of β-catenin reversed inflammatory response, EMT, and fibrosis progression mediated by SIK1 knockdown, which strongly revealing that WNT/β-catenin pathway was required for SIK1 mediated HK2 cells injury induced by AA.

### The Role of Twist1 in AA-induced HK2 cells injury

**Fig.8:**
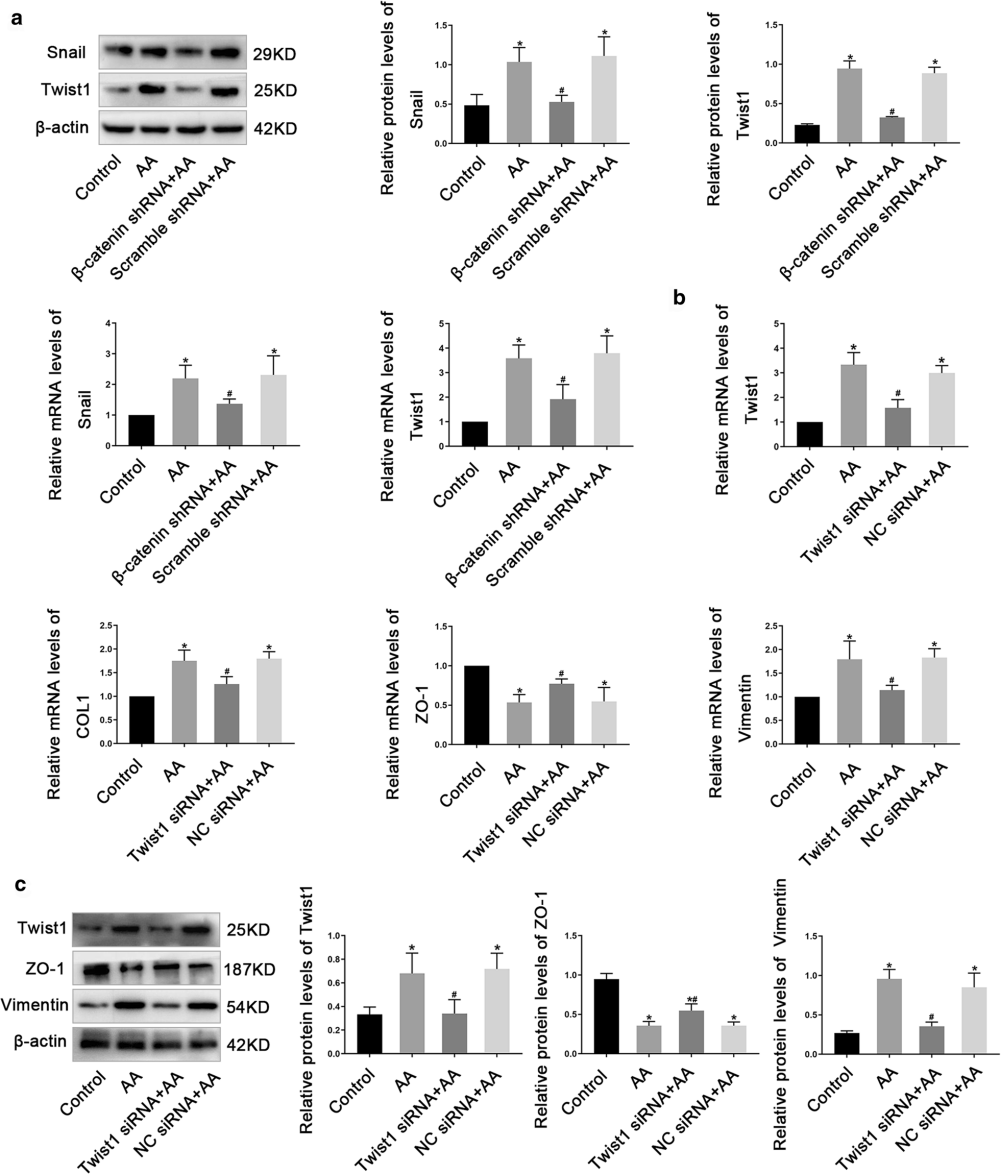
The role of Twist in AA-induced HK2 cells injury. **a** Western blot and real-time PCR analysis of Snail and Twist1 levels in HK2 cells. **b** real-time PCR analysis of Twist1, COL1, ZO-1 and Vimentin levels in HK2 cells treated with *Twist1* siRNA or NC siRNA. **c** Western blot analysis of Twist1, ZO-1 and Vimentin levels in HK2 cells treated with *Twist1* siRNA or NC siRNA. Data are shown as mean ± s.d. *P < 0.05 vs Control, ^#^*P* < 0.05 vs AA. All experiments were performed in triplicate

Acting as EMT-TFs, Twist1 can promote EMT and kidney fibrosis. In this study, we found AA promoted the protein and mRNA expression of Snail and Twist1 while knockdown β-catenin inhibited the expression of Snail and Twist1 induced by AA (Fig. 8a), indicating Snail and Twist1 located in the downstream of β-catenin and played a role in AA-induced HK2 cells injury. To verify our hypothesis, we knockdown Twist1 by siRNA (Additional file 7) and carried out real-time PCR and Western blot analysis. As expected, when compared with control cells stimulated with AA alone, the expression of Vimentin, and COLI was reduced while ZO-1 was increased in Twist1 siRNA cells in the presence of AA, suggesting silence of Twist1 alleviated the occurrence of EMT and the progression of renal fibrosis induced by AA (Fig. 8b and c).

## Discussion

AKI is a serious public health problem with high morbidity and mortality. In the past, AKI was considered to be reversible and a temporary decline in renal function. However, recent studies have gradually realized that the recovery of renal function in patients who survive AKI is often incomplete [23–25]. A meta-analysis has reported that patients with AKI had higher risks for developing CKD and ESDR, compared with patients without AKI [26]. Recently, the mechanism of AKI-CKD transition has attracted more and more attention from nephrologists. Many factors such as nephron loss, vascular insufficiency, endothelial injury, cell cycle disruption, interstitial inflammation and fibrosis, and maladaptive repair mechanisms may lead to the evolution of AKI into CKD [27]. The pathophysiological mechanisms are as follows: (1) After AKI, inflammatory cells release inflammatory factors and chemokines, and continuous inflammation can lead to the loss of renal function [28]; (2) Nephron loss, endothelial injury, vascular malfunction, leading to ischemia and hypoxia of renal tubular microenvironment and fibrosis of tubular interstitial [27]; (3) After AKI, tubular epithelial cell undergo EMT to produce myofibroblasts from the epithelia to heal the injured tissues. If the injury is mild and acute, the healing process is considered as reparative fibrosis; However, under continuous chronic inflammation, the abnormal formation of myofibroblasts can lead to progressive fibrosis, after which ECM accumulation and then the destruction of organ parenchyma will occur [29]; (4) The cell cycle G2/M was arrested in renal tubular epithelial cells after AKI, which can activate pro-fibrotic signaling pathway to induce profibrotic cytokine production [30]. In this study, we used AA to mimic the progression of AKI-CKD transition in vivo and in vitro, and we observed that AA stimulation can induce inflammation, EMT and fibrosis*,* which suggesting the successful establishment of AKI-CKD transition model.

Abundant evidences have demonstrated the role of SIK1 on EMT [8–10] and inflammation [11, 31], which are the hallmarks of AKI-CKD transition. For instance, it was reported that LKB1-SIK1 signaling pathway inhibited EMT by regulating the expression of some key transcription factors, including Snail2, Twist and ZEB1 [32]. More recently, the role of SIK1 in the kidney damage has drawn considerable attention. Ferrandi et al. have reported that nephrin and SIK1 co-localization in glomerular podocytes and there is a positive correlation between nephrin and SIK1 protein expression in rats and human renal specimens [12]. Moreover, SIK1 is involved in high glucose-induced mesangial cell proliferation and extracellular matrix accumulation mediated by the ALK5 signaling pathway [6]. All above indicate that SIK1 has a vital role in the kidney damage. SIK1 has a highly conserved serine (Thr182) in the kinase domain. After activated by the AMPK-activator LKB1 which phosphorylates SIK1 at Thr182, the activated SIK1 auto-phosphorylates its Ser186, and then maintains the sustained activity of SIK1 through sequential phosphorylation at Ser186-Thr182 by GSK-3β [33]. In this study, we discovered that the expression of SIK1 was downregulated in AKI patients and AKI mice, arousing our interest to further explore whether SIK1 was involved in AKI-CKD transition. By assessing the level of SIK1 in HK2 cells and C57BL/6 mice treated with AA, we observed that SIK1 and p-SIK1(Thr182) were down-regulated upon AA stimulation. The correlation of the decreased activity of SIK1 with its protein level under AA treatment is consistent with findings in HBZY-1 cells in which the level of Thr182 phosphorylation correlated with the SIK1 protein level under stimulation with high glucose [6]. What’s more, in the current study, we revealed the nuclear redistribution of SIK1 following AA stimulation in HK2 cells, which may result from the reduction in SIK1 kinase activity [6, 34, 35]. Further work is required to characterize the mechanism by which AA localizes SIK1 in the nucleus. Furthermore, we identified that overexpression of SIK1 delayed the progression of inflammation, EMT and fibrosis induced by AA both in vivo and in vitro. Thus, we concluded that AA induces AKI-CKD transition by inhibiting SIK1 and its phosphorylation level.

Multiple intracellular signal transduction pathways are involved in the expression and activation of EMT and renal fibrosis, including TGF-β signaling pathway, PI3K/AKT pathway, Src pathway, MAPK pathway, and WNT signaling pathway [36–41]. Among these, WNT/β-catenin signaling pathway, the most classic WNT pathway, was widely studied. Abundant data have suggested that WNT/β-catenin signaling plays a vital role in EMT [42, 43], inflammation [44, 45] and renal fibrosis [46–48]. β-catenin, a multifunctional protein, is the core molecular in the WNT signaling pathway. When there is no WNT signal stimulation, β-catenin is mainly connected to the proximal C-terminal domain of E-cadherin in the cell membrane; when stimulated by WNT signal, β-catenin translocates into the nucleus and binds to TCF/LEF transcription factors to stimulate the transcription of WNT target genes [49, 50]. The phosphorylation of β-catenin (Y654) leads to its release from E-cadherin protein and increases TCF-mediated transcriptional activity, balancing its role between cell adhesion and WNT signaling [51]. In addition, the increased phosphorylation level of β-catenin (Y654) can increase cell migration and induce tumor cell invasion [52]. In this study, we found that AA stimulation activated WNT/β-catenin signaling pathway and enhanced transcriptional activity of TCF and LEF. In addition, we observed knockdown of β-catenin alleviated the inflammatory response, EMT and fibrosis induced by AA, suggesting that WNT/β-catenin signaling pathway is involved in AKI-CKD transition, which was consistent with a previous study [17]. Furthermore, we discovered SIK1 regulated WNT/β-catenin signaling pathway both in vivo and in vitro, further supporting the role of SIK1 in AA-induced AKI-CKD transition.

EMT and renal fibrosis require a powerful transcription mechanism to regulate. The transcription factors that activate EMT and fibrosis are mainly divided into three major groups: Snail transcription factors, ZEB transcription factors and bHLH transcription factors. Snail is a zinc finger protein that acts as a transcriptional repressor by recognizing the E-box in the promoter of the target gene and the increased expression of Snail is involved in EMT [53]. Similar to the effect of Snail, Twist1 downregulated the expression of epithelial phenotype-related genes and induced the expression of mesenchymal phenotype-related genes [54]. The role of Snail and Twist1 in the process of AA-induced AKI-CKD transition is still not fully understood. In this study, we found AA promoted the protein and mRNA expression of Snail and Twist1 while knockdown β-catenin inhibited the expression of Snail and Twist1 induced by AA. Furthermore, we observed that silenced Twist1 by siRNA alleviated the occurrence of EMT and the progression of renal fibrosis induced by AA, suggesting Twist1 plays an important role in AA-induced AKI-CKD transition. Further studies are required to determine whether SIK1 could regulate the expression of Twist1 during AKI-CKD transition.

## Conclusion

**Fig.9:**
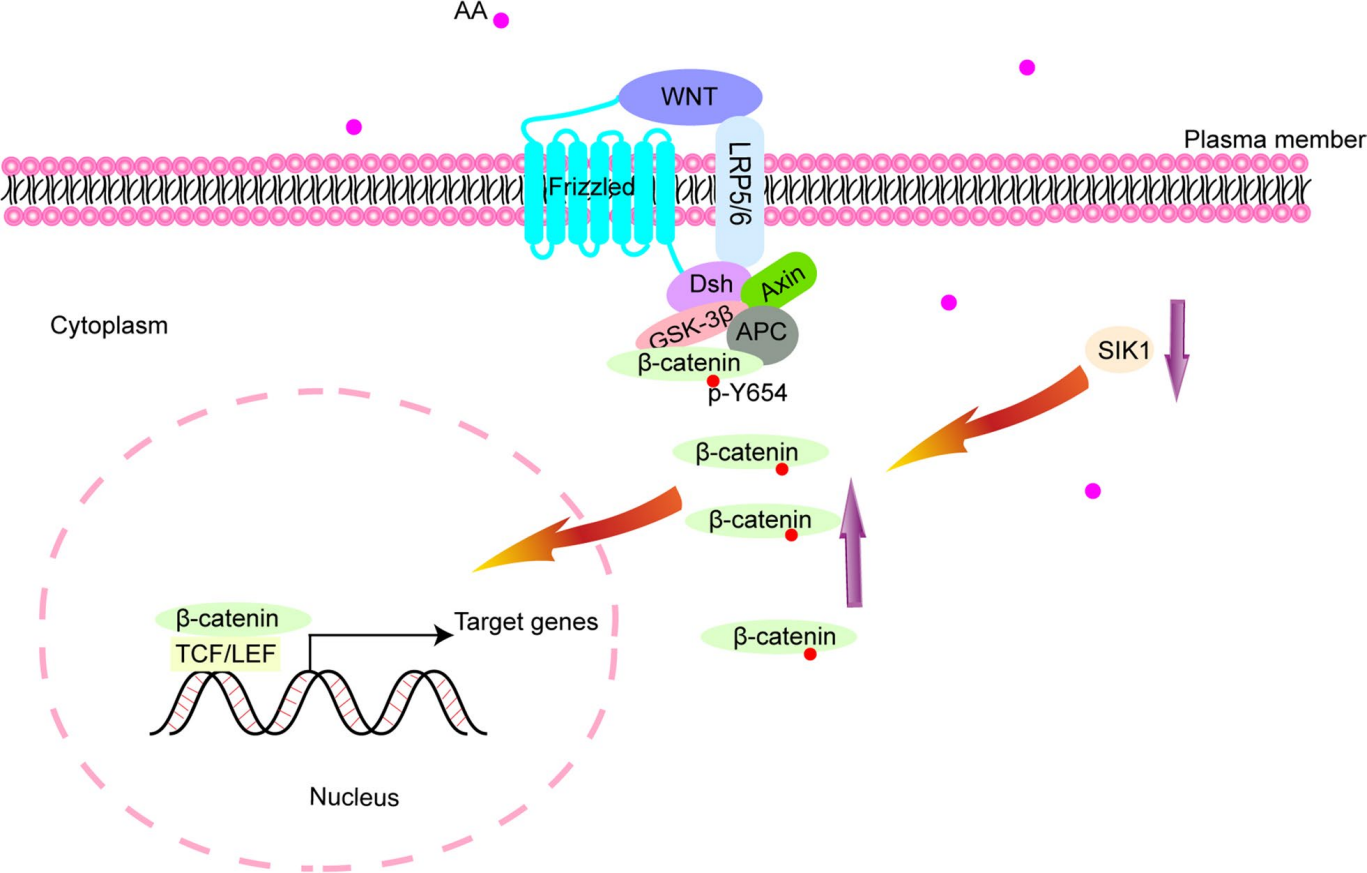
Schematic model of the role of SIK1.AA stimulation down-regulated the expression of SIK1, leading to up-regulation of p-β-catenin (Y654) and nuclear accumulation of β-catenin, thus promoting the transcription of target genes by activating the WNT/β-catenin signaling pathway

In this study, we demonstrated that SIK1 was involved in the AA-induced AKI-CKD transition, and we showed that SIK1 participated in AKI-CKD transition through WNT/β-catenin signaling pathway (Fig. 9). Upregulation of SIK1, or inhibition of WNT/β-catenin signaling pathway alleviate the inflammation, EMT, and fibrosis induced by AA, delaying the progression of AKI-CKD transition. These observations will provide a new therapeutic target for the clinical prevention and treatment of renal fibrosis after AKI.

## Supplementary Information

**Additional file 1. a** Schematic diagram of GV467 carrier information. **b** The ability of AAV9 virus vector to transduce 293 T cells. The high-content imaging system showed that the positive rate of EGFP green fluorescence protein in the AAV9-*Sik1* transfection group. Magnification 200 × . **c** The ability of AAV9 virus vector to transduce kidney of C57BL/6 mice by tail vein injection. Scale bar = 200 μm.

**Additional file 2.** HK2 cells were treated with different concentrations of AA (10, 20, 40, and 60 µmol/L) for 72 h, and CCK8 were performed to detect the viability of HK2 cells. Data are shown as mean ± s.d. *P < 0.05 vs Control. All experiments were performed in triplicate.

**Additional file 3.** Representative immunofluorescence images of SIK1 in HK2 cells treated with 10 μmol/L AA for 0 h, 24 h, 48 h and 72 h. Scale bar = 50 μm.

**Additional file 4.** The overexpression efficiency of SIK1. HK2 cells were treated with SIK1 lentiviral overexpression vector (SIK1 vector) or Control vector, the overexpression efficiency was examined by real-time PCR(**a**) and Western blot (**b**). Data are shown as mean ± s.d. *P < 0.05 vs Control. The experiment was performed in triplicate.

**Additional file 5.** The knock-down efficiency of SIK1. HK2 cells were treated with *SIK1* lentiviral shRNA (*SIK1* shRNA) or Scramble shRNA, the knock-down efficiency was examined by Western blot. (**a**) and real-time PCR (**b**). Data are shown as mean ± s.d. *P < 0.05 vs Control. ^#^*P* < 0.05 vs AA. The experiment was performed in triplicate.

**Additional file 6.** AA stimulation can activate WNT/β-catenin signaling pathway in HK2 cells. **a** Western blot analysis of WNT1, nuclear β-catenin and p-β-catenin (Y654) levels in HK2 cells. **b** Representative immunofluorescence images of β-catenin in HK2 cells. Scale bar = 50 μm. **c** HK2 cells were treated with β-catenin lentiviral shRNA (β-catenin shRNA) or Scramble shRNA, the knock-down efficiency was examined by real-time PCR and Western blot. Data are shown as mean ± s.d. *P < 0.05 vs Control, ^#^*P* < 0.05 vs AA. The experiment was performed in triplicate.

**Additional file 7.** The knock-down efficiency of Twist1. HK2 cells were treated with *Twist1* siRNA or NC siRNA, the knockdown efficiency was confirmed by real-time PCR (**a**) and Western blot (**b**). Data are shown as mean ± s.d.*P < 0.05 vs Control. The experiment was performed in triplicate.

## Data Availability

All data generated or analyzed during this study are included either in this article or in the Additional files.

## Abbreviations

AKI: Acute kidney injury
CKD: Chronic kidney disease
SIK1: Salt inducible kinase 1
AA: Aristolochic acid
GFR: Glomerular filtration rate
EMT: Epithelial-mesenchymal transition
AMPKs: AMP-activated protein kinases
ECM: Extracellular matrix
Scr: Serum creatinine
BUN: Blood urea nitrogen
HK2: Human proximal tubular epithelial cells
FBS: Fetal bovine serum
NSCLC: Non-small cell lung cancer
