# Supplementary figures and images for: Role of SIK1 in the transition of acute kidney injury into chronic kidney disease
Source: J Transl Med. 2021 Feb 15;19:69. doi: 10.1186/s12967-021-02717-5 (PMC7885408; doi:10.1186/s12967-021-02717-5)

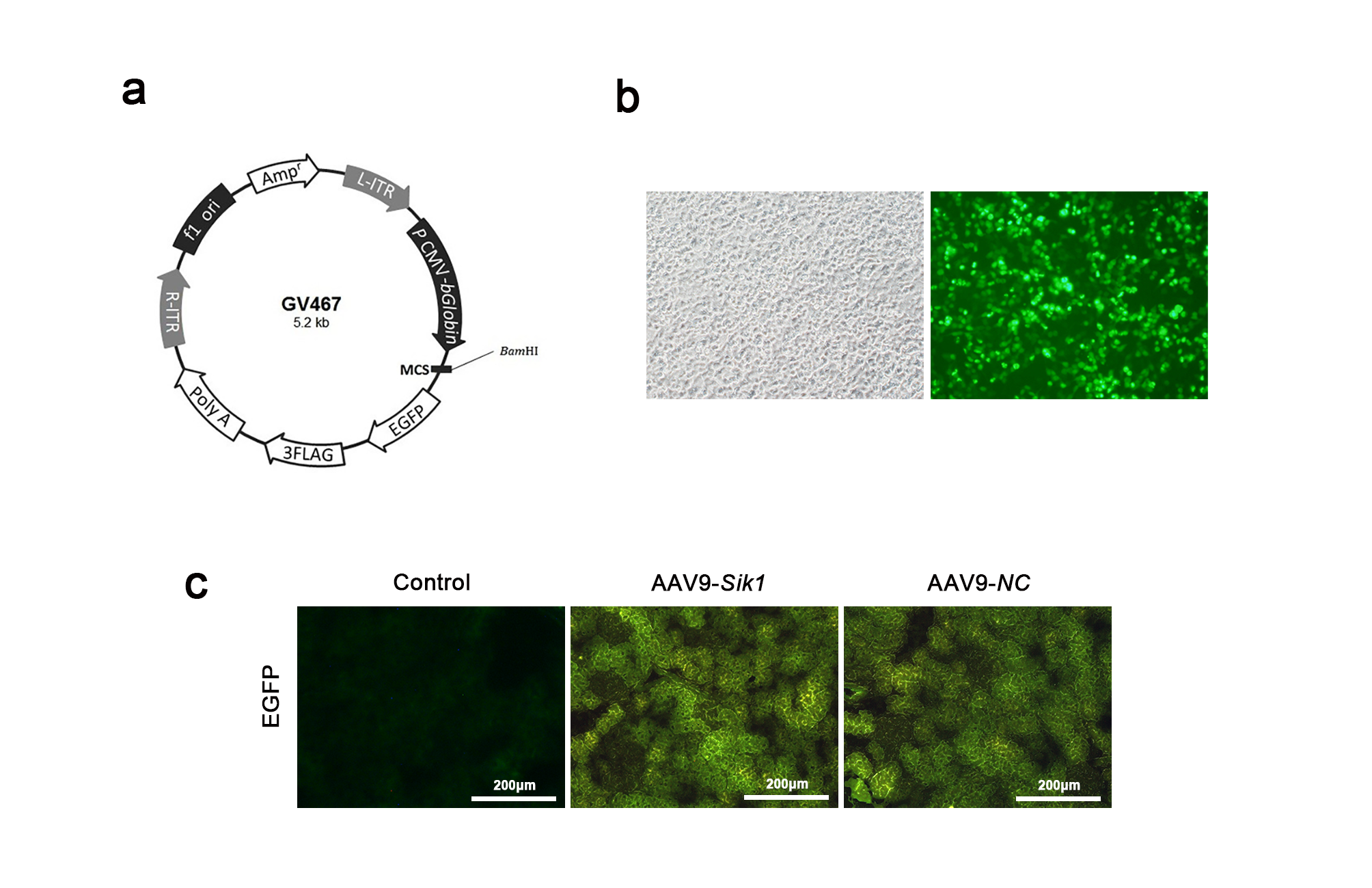

Supplement: Supplementary file 1 — Additional file 1. a Schematic diagram of GV467 carrier information. b The ability of AAV9 virus vector to transduce 293 T cells. The high-content imaging system showed that the positive rate of EGFP green fluorescence protein in the AAV9-Sik1 transfection group. Magnification 200 × . c The ability of AAV9 virus vector to transduce kidney of C57BL/6 mice by tail vein injection. Scale bar = 200 μm. [file 12967_2021_2717_MOESM1_ESM.tif]

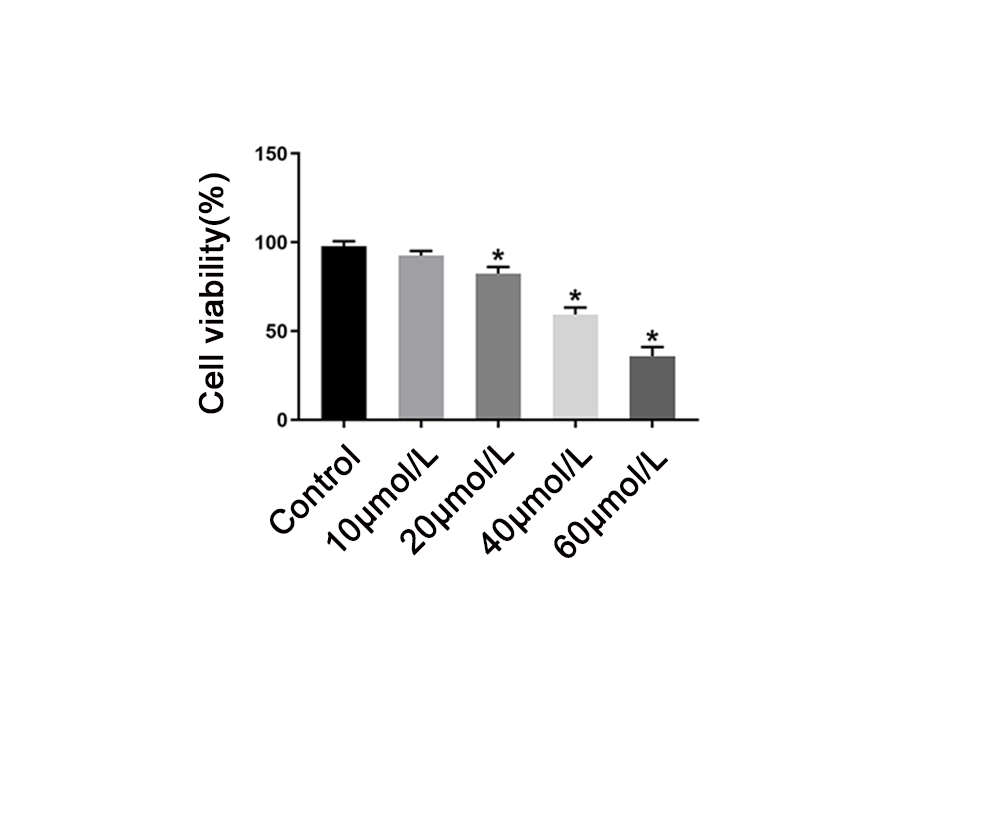

Supplement: Supplementary file 2 — Additional file 2. HK2 cells were treated with different concentrations of AA (10, 20, 40, and 60 µmol/L) for 72 h, and CCK8 were performed to detect the viability of HK2 cells. Data are shown as mean ± s.d. *P < 0.05 vs Control. All experiments were performed in triplicate. [file 12967_2021_2717_MOESM2_ESM.tif]

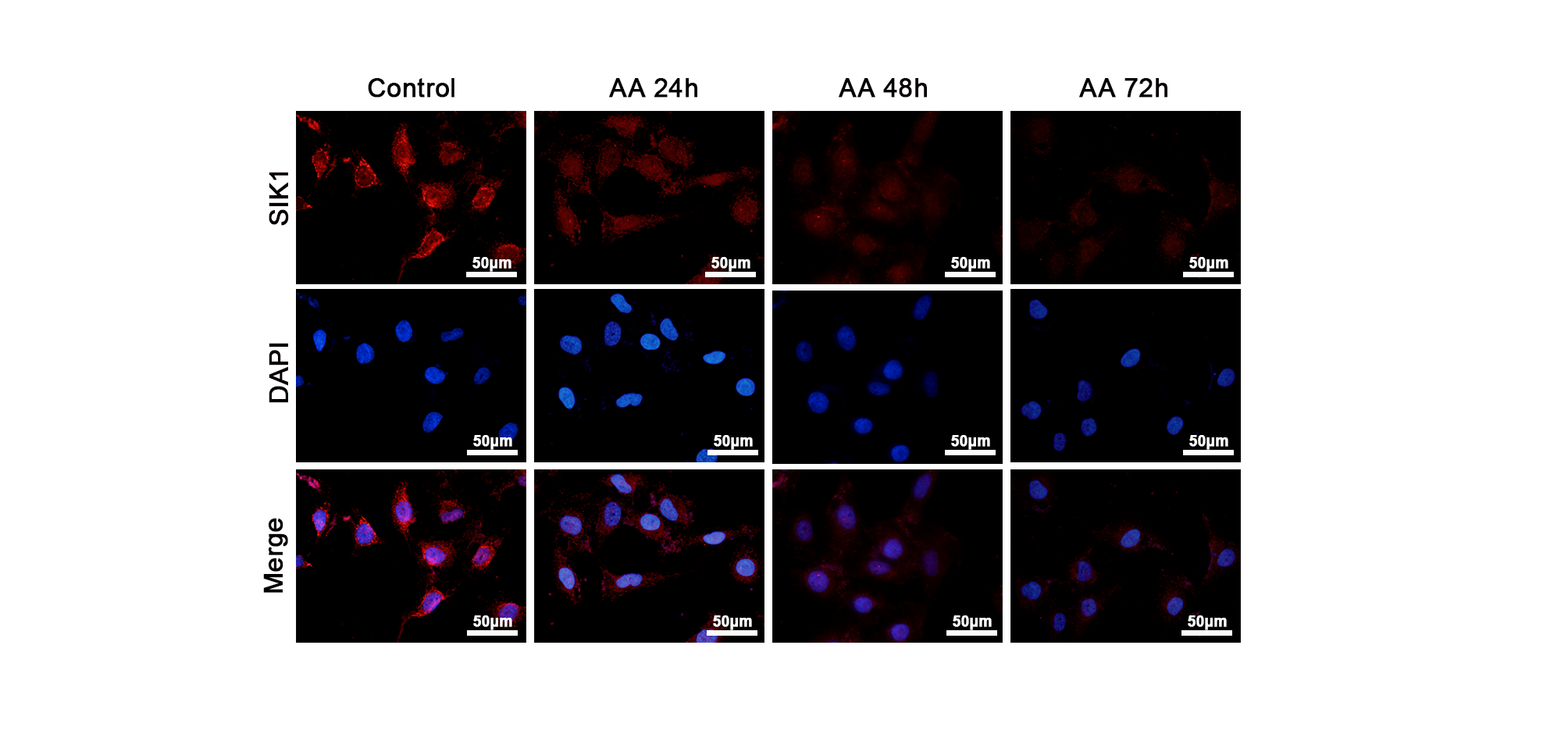

Supplement: Supplementary file 3 — Additional file 3. Representative immunofluorescence images of SIK1 in HK2 cells treated with 10 μmol/L AA for 0 h, 24 h, 48 h and 72 h. Scale bar = 50 μm. [file 12967_2021_2717_MOESM3_ESM.tif]

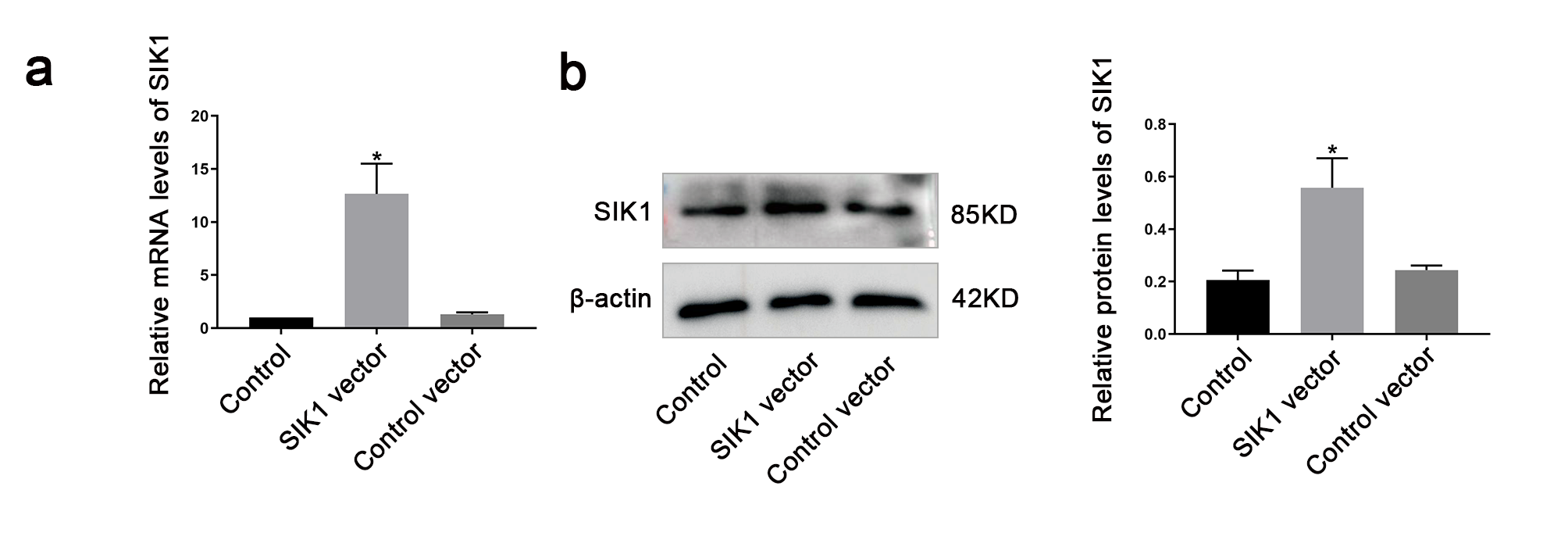

Supplement: Supplementary file 4 — Additional file 4. The overexpression efficiency of SIK1. HK2 cells were treated with SIK1 lentiviral overexpression vector (SIK1 vector) or Control vector, the overexpression efficiency was examined by real-time PCR(a) and Western blot (b). Data are shown as mean ± s.d. *P < 0.05 vs Control. The experiment was performed in triplicate. [file 12967_2021_2717_MOESM4_ESM.tif]

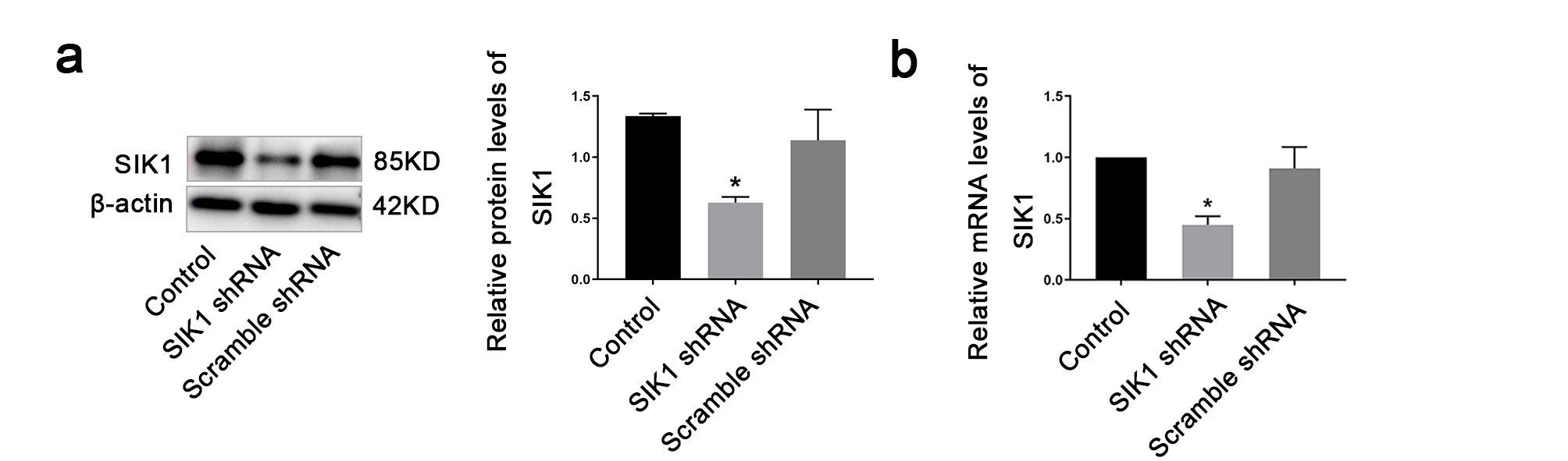

Supplement: Supplementary file 5 — Additional file 5. The knock-down efficiency of SIK1. HK2 cells were treated with SIK1 lentiviral shRNA (SIK1 shRNA) or Scramble shRNA, the knock-down efficiency was examined by Western blot. (a) and real-time PCR (b). Data are shown as mean ± s.d. *P < 0.05 vs Control. #P < 0.05 vs AA. The experiment was performed in triplicate. [file 12967_2021_2717_MOESM5_ESM.tif]

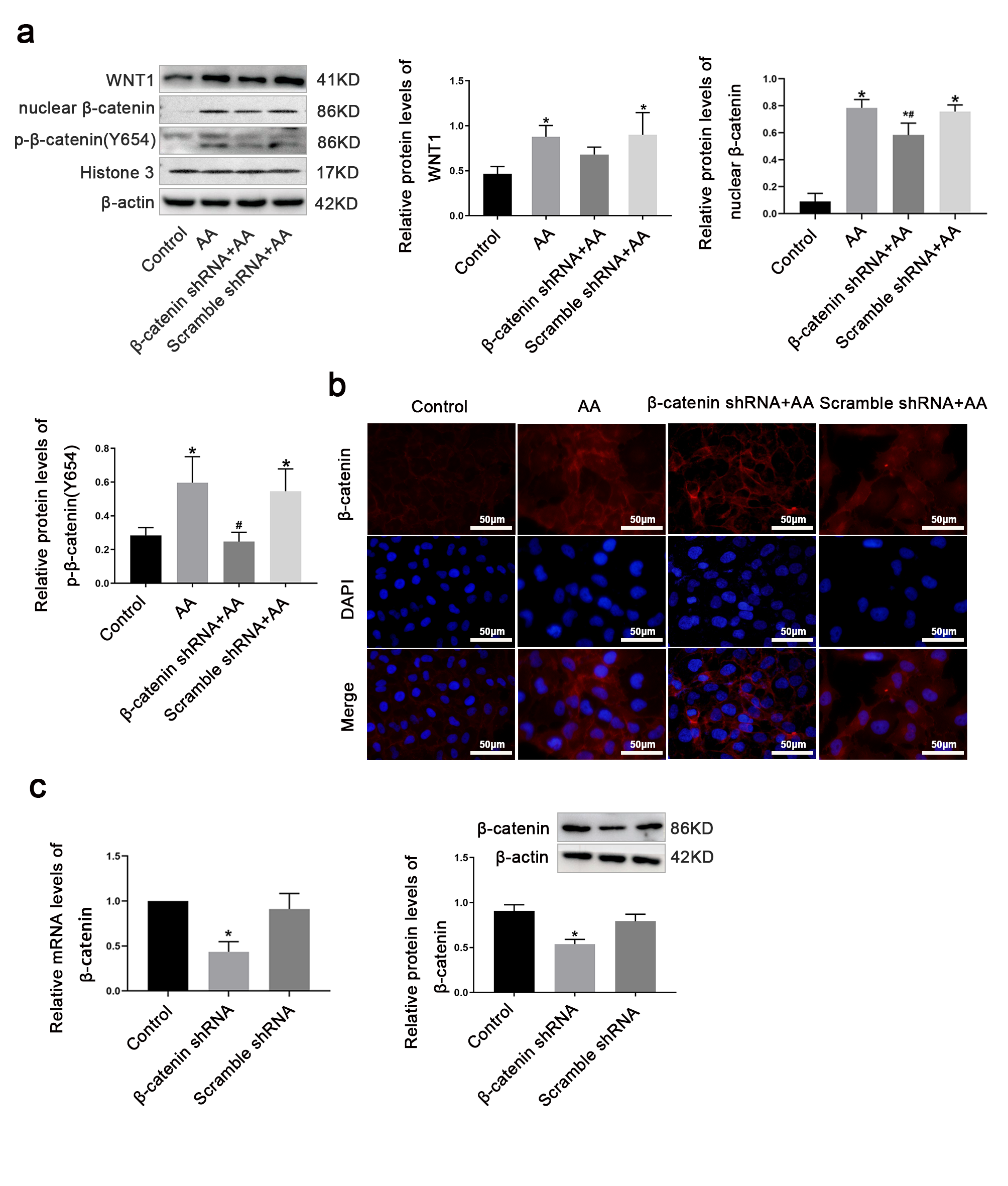

Supplement: Supplementary file 6 — Additional file 6. AA stimulation can activate WNT/β-catenin signaling pathway in HK2 cells. a Western blot analysis of WNT1, nuclear β-catenin and p-β-catenin (Y654) levels in HK2 cells. b Representative immunofluorescence images of β-catenin in HK2 cells. Scale bar = 50 μm. c HK2 cells were treated with β-catenin lentiviral shRNA (β-catenin shRNA) or Scramble shRNA, the knock-down efficiency was examined by real-time PCR and Western blot. Data are shown as mean ± s.d. *P < 0.05 vs Control, #P < 0.05 vs AA. The experiment was performed in triplicate. [file 12967_2021_2717_MOESM6_ESM.tif]

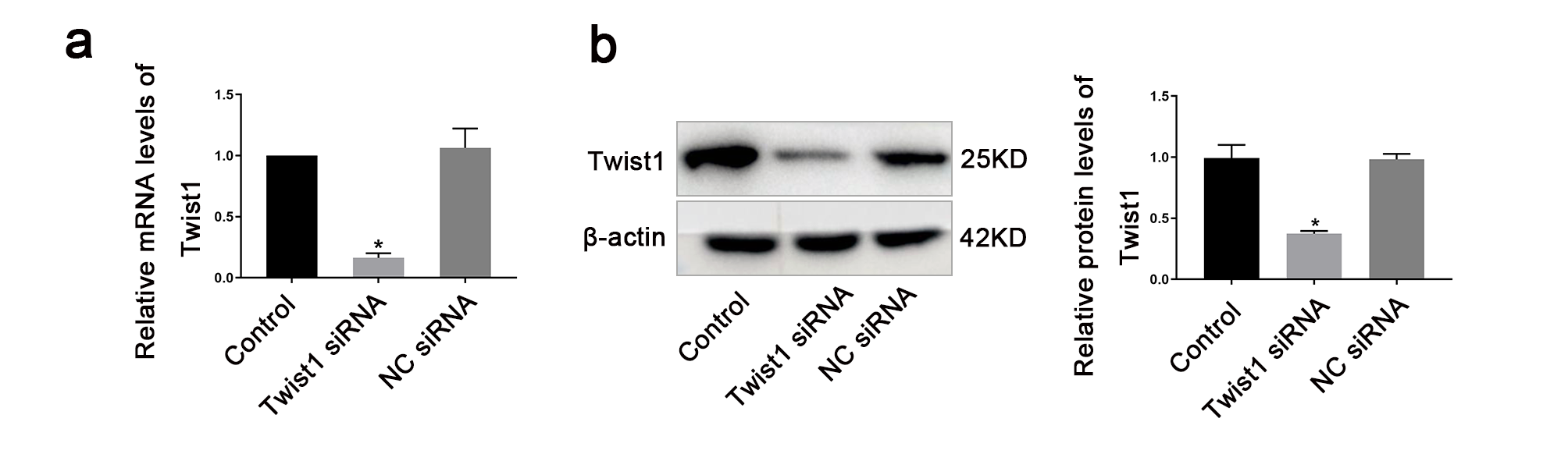

Supplement: Supplementary file 7 — Additional file 7. The knock-down efficiency of Twist1. HK2 cells were treated with Twist1 siRNA or NC siRNA, the knockdown efficiency was confirmed by real-time PCR (a) and Western blot (b). Data are shown as mean ± s.d.*P < 0.05 vs Control. The experiment was performed in triplicate. [file 12967_2021_2717_MOESM7_ESM.tif]
